# Peritonsillar Abscess

**Published:** 1898-07

**Authors:** Dunbar Roy

**Affiliations:** Professor Ophthalmology and Otology in Southern Medical College, Atlanta, Ga.


### PERITONSILLAR ABSCESS.

By DUNBAR ROY, A.B., M.D.,

Professor Ophthalmology and Otology in Southern Medical College, Atlanta, Ga.

The reading of this article is more for the purpose of precipitating a discussion than an effort to add any new ideas upon the subject. A great deal has been written by laryngologists concerning the etiology, symptoms and treatment of acute tonsillitis and chronically hypertrophied tonsils, but very little can be found concerning the manifestations of peritonsillar abscess; and even the various text-books dismiss this practical subject with but few words, if at all. Fletcher Ingalls in his excellent work denominates this condition as phlegmonous tonsillitis, giving as synonyms

"suppurative tonsillitis," "abscess of the tonsils," "quinsy," "phlegmonous sore throat." To my mind these synonymous terms are incorrect, for in my experience the abscess is nearly always outside of the tonsil. Phlegmonous sore throat is inaccurate because it does not designate what portion of the throat is involved. The practical point of opening the abscess is dismissed with one sentence. Sajous in his text-book makes no distinction in the forms of tonsillitis but considers them all together and the condition of abscess of the tonsil but a final result of acute tonsillitis. He also calls it an abscess of the tonsil and speaks of opening the abscess through the tonsil.

Lennox Browne, while recognizing this condition, devotes very little space to its consideration. He remarks that Cohen uses the term "phlegmonous pharyngitis" as synonymous with tonsillitis, but remarks "that the peritonsillar tissue is affected rather than the gland itself in phlegmonous inflammation."

Max Thorner in Burnett's System has given a very good description of this affection under the title of phlegmonous pharyngitis.

Dr. C. E. Bean in the same work has also described this affection under the title of acute tonsillitis.

Thus we see various writers differ as to the pathology and morbid anatomy of this affection.

The condition which I wish to speak of to-day I have designated as peritonsillar abscess. Phlegmonous pharyngitis is, I think, an inelegant term, since it does not indicate the anatomical position of the abscess and therefore does not differentiate it from similar conditions in other portions of the pharynx.

The tonsils are lymphoid structures situated between the palatine arches, the anterior and the posterior. They are freely movable in their bed and in the large majority of cases they present fibrous adhesions to both the anterior and posterior palatine pillars. The muscular structures therefore inclose the tonsil on all sides except its free portion. In front the ramus of the lower jaw encroaches so near the tonsil that there is very little loose cellular space left which could be filled with pus and exudate going to form an abscess. Pus always seeks that region where there is the less resistance. On the other hand the anterior pillar curves around the top of the tonsil and seems to lap itself over the posterior pillar and is lost in the soft palate above. This is what really happens. Posteriorly to the tonsil there is quite a loose cellular space which could be filled with pus should the emergency come. This lapping of the anterior and posterior pillar above seems to have formed a triangular space filled with connective tissue and into which the tonsil communicates.

For these anatomical reasons I have never seen a peritonsillar abscess the result of infection through the tonsillar lacunæ, but what had its seat posterior to the tonsil. Abscesses which occur anteriorly are, in my experience, the result of suppurative processes from the posterior portion of the alveolar process of the lower or upper jaw on the same side. I have seen abscesses in front of the tonsil of the most painful character which were the result of a process in the last molar tooth.

The question now arises what is the cause of these peritonsillar abscesses? Some authors hold that they are always the result of a previous tonsillitis.

In the light of our present knowledge there can be no doubt in my mind as to the bacterial origin of this affection. It is certainly true that in the large majority of cases a severe inflammation of the tonsil precedes the formation of the peritonsillar abscess, and yet I have seen cases where the abscess has formed and the tonsil remain seemingly scarcely involved. We all know that there exists normally in the mouth and throat secretions containing various kinds of micro-organisms, therefore when a tonsillitis does take place there must be superadded something which previously did not exist and much the more so when an abscess is formed. For it is the daily observation to see a case of severe tonsillitis of the most virulent type and yet no abscess is seen to follow. Some authors say that it is due at the time to some lowered vitality of the parts of the whole system whereby the parts are unable to throw off this concentrated poison. Observation certainly seems to contradict this idea unless there is some lowered vitality of the parts not now recognizable to the medical eye, for the worst cases of abscesses which I have ever seen have occurred in persons showing the most perfect physical health.

The tonsillar crypts and ducts are certainly the carriers of the deadly organisms, for in the large majority of cases these crypts show follicular exudates previous to or at the time the abscess develops. Whatever may be the condition of the parts, one thing is certain and that is this affection is microbic in origin and the warfare between the leucocytes and the pus-forming organisms certainly results in a victory for the latter.

Must we not in the end be obliged to admit that there exists a peculiar idiosyncrasy in the individual which we are unable to explain whereby his or her throat is liable to the formation of peritonsillar abscesses? One attack seems to predispose to another, and there are some people who seem especially liable to just such attacks. I have one patient who usually has one severe attack every winter, yet when the attacks are over the tonsils do not show any pathological changes.

Is it not perhaps the existence of this triangular space of which I have spoken that predisposes to the formation of an abscess? I have always noticed that in persons who suffer from these abscesses the anterior pillar seems to lap more than ever the posterior, and it is but a surmise on my part that in these cases there is a larger posterior cellular space for the filling up with pus.

Rice, of New York, in the Cincinnati *Lancet-Clinic*, in a very exhaustive and excellent article on suppurative tonsillitis, has this to say in his summary:

"That suppurative tonsillitis is not a correct name because the suppuration occurs in the connective tissue about the tonsils and very rarely in the tonsils themselves. That in people who possess the disposition to suppuration about the tonsils, we find the tonsils either adherent to or covered by the pharyngeal pillars and that this condition plays a more important rôle in the predisposition to suppuration about the tonsil than does the rheumatic or other diathesis."

Such a space affords an excellent bed for the propagation of pus germs. Nor have I ever seen any special character of tonsil which seems to predispose the formation of an abscess. I have seen abscesses form where tonsils were small and sclerosed and again where they were large and lymphoid looking. I have seen abscesses form when the tonsil had been completely removed.

Most writers hold that peritonsillar and tonsillar abscesses are more common in children than in adults, and yet if one's experience counts for anything, mine is just the opposite. With the exception of two cases only all my cases have been in adults.

The symptoms of this condition are no doubt familiar to all. The pain is severe and especially intense on deglutition, at which moment the patient distorts his face, makes forced movements with his whole body in his effort perhaps to swallow even a drop of saliva. This pain is pathognomonic, and even in the very earliest stages is often indicative of the coming abscess. The pain is due to the inflammatory condition of the muscles of deglutition, and it is for this reason I think that so many authors believe that a rheumatic diathesis is at the bottom of it all. This view my observation does not lead me to accept.

The coated tongue, the fetid breath, the half open mouth, the look of pain, who does not recognize these symptoms?

Let us now turn to the practical side of the question, that is of its treatment. Since, as I have said, the large majority of cases result from a previous inflammation of the tonsil, the question naturally arises, can the abscess be aborted? My views are that, if the patient is not one of those predisposed to peritonsillar abscess, and the inflammation in the tonsil has started upon its surface, you may in some cases abort the abscess, but in the large majority of cases you are powerless to stay its course. The treatment of tonsillitis and also peritonsillar abscess naturally divides itself into the medicinal and the surgical. Under the head of medicinal let us look to see what remedies are suggested by various authors as abortive or curative in their action.

Ingalls says, to use his exact words, "early in the attack the disease (phlegmonous tonsillitis) may be aborted as in acute tonsillitis in about one case out of four by the application to the inflamed gland once or twice a day, of a sixty-grain solution of nitrate of silver, two or three applications usually being sufficient." Of this procedure I can but speak in the very highest terms in all forms of acute tonsillitis, and in my mind it should be placed above all other remedies. Dr. Ingalls's experience with guaiac is just as mine has been, and that is very unsatisfactory. He believes in sodium salicylate and bromide of potash. He recommends cold applications both inside and outside, but also remarks that some patients are made uncomfortable by cold applications and then hot ones should be applied. He believes in promoting resolution if the abscess cannot be aborted.

Sajous says that we possess a remedy which is a specific in all forms of tonsillitis, and that is the ammoniated tincture of guaiac. He prescribes it as follows: one teaspoonful in half glass of milk to be gargled in the throat and then swallowed. He also recommends lozenges of the resin of guaiac. He recommends a gargle of water, hot as can be borne, a procedure which I have long ago found to be a most excellent adjuvant for the relief of pain. As to the use of guaiac I can but say that in my hands it has proved absolutely useless.

Lennox Browne considers guaiac to be almost a specific, and he says further that this action of guaiac "strengthens the rheumatic analogy." He is a great believer in large doses of salicylic acid. He says that sprays and inhalations are useless while ice internally aggravates the pain. Like myself he believes in hot gargles of some mild antiseptic solution. He considers the application of cold externally by means of the Leiter coil one of the most valuable local applications.

Bosworth thinks "that it is possible to abort an attack within the first twenty-four hours by giving 10 grains of quinine and 1 grain opium, administering a hot foot-bath, evacuating the bowels by 15 grains of calomel to be followed by a saline purgative, and giving sodii salicylas and applying locally to the throat sodii bicarb." I must say that this is a most drastic remedy, and if the patient survives it, nothing short of a cure should be his reward.

Max Thorner recommends the administering of large doses of salol in addition to local applications. I am in full accord with this writer who expresses not much belief in cold applications but says "it is much better to begin early with hot fomentations around the neck, hot gargles and hot inhalations, in order to accelerate suppuration and to shorten in this way the duration of the disease."

Dr. C. B. Bean, in Burnett's System, says that he places more confidence in salicylate of soda than any other remedy. He says that usually permanent relief will be obtained in twelve hours. He administers it in doses of 15 grains every hour.

Gougueheim of Paris, has extolled the use of large doses of salol.

Let us now look to the surgical side of the treatment where the abscess is either suspected to have formed or at least shows all evidences of the same. I will first take the liberty of again quoting the opinions of those who have expressed themselves, and in conclusion will add my own practical ideas to this practical subject.

Ingalls says "pus should be evacuated as soon as discovered." He does not say where the incision should be made, how deep it should go, nor the most usual place for the bulging to occur. These points I consider of very practical value.

Sajous says that the abscess should be evacuated as soon as it is discovered by digital pressure and the incision is made through the tonsil.

Lennox Browne lays down the following rules which I quote:

1. Never to inflict unnecessary pain by useless scarifications on the surface of the tonsil undergoing inflammation.

2. Never to make deep incisions unless there is almost certainty of advanced suppuration. The instrument for making the incisions should be a curved-pointed bistoury with not more than one inch of cutting edge, and the cut should be made from without inwards, so as to avoid the not impossible risk of injuring an artery.

Bean says the abscess should be opened as soon as pus is detected, and that it should be done with a bistoury whose cutting edge is an inch long. He says also that the point for opening the abscess is usually "at the upper and anterior surface of the tonsil near the anterior palatine fold."

My observations certainly do not agree with this latter statement. Max Thorner also says that the abscess should be opened as soon as discovered, and gives the method of Stoerk for diagnosing the condition. The physician "puts the fingers of one hand externally under the angle of the lower jaw, pressing the skin and all the tissues inwardly, while the index-finger of the other hand moves slowly over the infiltrated parts, beginning high up on the soft palate and sliding downwards towards the tongue." The sense of resistance is the criterion for the point to be opened. This is very good for a German method but it is decidedly too rough for an American patient, as it frequently happens that you are fortunate if you can just get in the flat blade of a bistoury between the half-open mouth.

Moure of Bordeaux says "it is the rule to-day not to wait for the appearance of pus before incising the inflamed tissues." He says further that a peritonsillar abscess should be opened through the anterior pillar by a longitudinal incision.

O. Chiari also advises early incision, even when no pus is discovered and says the abscess should be opened at the anterior pillar. My own experience certainly does not agree with that of the last two writers.

Gougueheim of Paris, deprecates too hearty operative measures in phlegmonous tonsillitis. He admits surgical intervention only in exceptional cases, and that is when the appearance of whitish transparent spots reveals the purulent focus.

My own treatment of peritonsillar abscess is governed by a rule which I have adopted in the treatment of all patients, and that is the adapting of all treatment to the individual case and not the patient to the treatment. It is the extreme of folly for physicians to have fixed rules in the management of all cases. It might be highly proper in the case of a big strong lymphatic working man to pick up your bistoury and plunge it into the abscess, press its sides and evacuate the same thoroughly, and he, perhaps, would hardly flinch. But suppose you undertook the same procedure in the case of a highly nervous and sensitive woman who would even faint at the sight of a knife. Besides it is equally true that the formation of a peritonsillar abscess is not near so painful in some patients as in some others, and therefore do not require such active surgical measures. As the majority of abscesses follow from tonsillitis, my efforts are always to the abortion of this inflammation and this I accomplish if at all, by the administration of a good dose of calomel followed by a saline purge. The tonsil and surrounding pillars are painted thoroughly with a 60-grain solution of nitrate of silver, and this is repeated once daily. I start early with hot gargles of vinegar and hot water, as hot as can be borne, and then hot fomentations are applied externally. Salol and phenacetin always makes the patient feel more comfortable, and for this reason alone I prescribe it.

Recently we have all seen the published reports of the wonderful effect of lactophenin in this condition. According to my experience it is no more good than so much sugar of milk. If, then, this does not abort the abscess, I increase the use of the hot remedies and wait until I detect fluctuation. In nervous and timid individuals I never try to open the abscess until it is so superficial that the slightest puncture will rupture its walls. Making deep incisions at random, without finding pus, is an unnecessary and barbarous treatment.

Nor have I ever opened a peritonsillar abscess at any point except above and posterior to the tonsil, notwithstanding such eminent authority as quoted above. Nor, furthermore, do I make deep incisions with a knife, but simply make a small puncture about one-eighth inch deep, and then, with a strong probe in this opening, I push it back into the triangular space described. If there is pus the probe will readily make its way, and the presence of pus be detected at the opening. If present, the superficial incision is enlarged and the abscess emptied by pressure. I do this rather than make a deep incision, because I have seen such an incision followed by severe hemorrhage and no pus found; and if the patient is at all nervous, be it man or woman, the sight of spitting out great clots of blood is extremely terrorizing. While writing this article I came across two references in the *Laryngoscope* to articles by German laryngologists, whose ideas corresponded with mine. I quote the exact words translated from Killian's article: "Most of these abscesses are located in the depression above the tonsil (*fossa supratonsillaris*) and between the arches of the palate. To inspect this region the patient's tongue is extended and depressed, the commissure of the lip is contracted, and the head inclined towards the shoulder of the diseased side. In an abscess of any considerable size one can see an oval bulging of the affected side.

This area is cocainized, and, by pressure upwards and outwards with a strong probe, the abscess cavity can be reached. The opening is then dilated with forceps for several days and antiseptic gargles ordered." Grunwald, in the *Munchener Med. Woch.*, indorses this method and insists that an abscess that cannot be reached in this way has no connection with the tonsil. In support of this view he reports an abscess of the anterior palatine arch and velum, due to a decayed molar tooth and its alveolus. This last view corresponds so entirely with my own that I feel like taking it as my own utterance. If any amount of pus has formed, noted by a swelling above and to the inner side of the tonsil, I can usually detect the point for opening by simply sliding a blunted probe over the surface and noting the point of greatest pain. My views correspond with those of Lennox Browne, and that is never to do unnecessary cutting.

The question also arises, if the abscess is not opened will it open itself? It most assuredly would, but I have never waited for such a result to happen. In the first place, it might open while the patient was asleep and cause some dangerous symptoms. Such cases have been reported. Dr. Dunn, of Richmond, Va., has reported the case of a child three and a half years old where a peritonsillar abscess broke spontaneously, followed by such severe hemorrhage that the common carotid had to be ligated. Norton has reported a case of acute suppurative tonsillitis in a girl of four years, which ended fatally, the abscess having involved the carotid.

Thus we see that peritonsillar abscesses are by no means an insignificant affair. Every physician no doubt manages his cases differently, but after all we want to use those measures which will bring the best results.

*Grand Opera House.*

---

A few drops of strong ammonia water added to the water in which you wash will greatly facilitate the removal of grease and blood. About half a teaspoonful to an ordinary basin of water will be the right proportion. Of course you must use soap as well.—*International Journal of Surgery.*

